# Ultrasensitive nano-aptasensor for monitoring retinol binding protein 4 as a biomarker for diabetes prognosis at early stages

**DOI:** 10.1038/s41598-019-57396-6

**Published:** 2020-01-17

**Authors:** Raheleh Torabi, Hedayatollah Ghourchian

**Affiliations:** 1 Laboratory of Bioanalysis, Institute of Biochemistry and Biophysics, University of Tehran, Tehran, Iran; 2 Nanobiomedicine Center of Excellence, Nanoscience and Nanotechnology Research Center, University of Tehran, Tehran, Iran

**Keywords:** Biological fluorescence, Nanoscale materials

## Abstract

Prognosis of diabetes risk at early stages has become an important challenge due to the prevalence of this disease. Retinol binding protein 4 (RBP4), a recently identified adipokine, has been introduced as a predictor for the onset of diabetes type 2 in coming future. In the present report a sensitive aptasensor for detection of RBP4 is introduced. The immune sandwich was prepared by immobilizing biotinylated RBP4 aptamers on streptavidin coated polystyrene micro-wells and then incubation of RBP4 as target and finally addition of luminol-antibody bearing intercross-linked gold nanoparticles as reporter. The chemiluminescence intensity was recorded in the presence of hydrogen peroxide as oxidant agent and Au^3+^ as an efficient catalyst for luminol oxidation. The aptasensor responded to RBP4 in the linear concentration range from 0.001 to 2 ng/mL and detection limit was slightly less than 1 pg/mL. The proposed method has successfully applied to determine the RBP4 in patient real serums. By using the intercross-linked gold nanoparticles, it is possible to provide more accessible surface for immobilizing luminol and enhance the chemiluminescence signal. Therefore, the analytical parameters such as sensitivity, specificity, detection limit and linear range were improved in compare to the biosensors reported in the literature.

## Introduction

Type 2 diabetes (T2D) has become a major challenge for public health worldwide. Increasing the prevalence of T2D could be due to the factors such as aging, urbanization, obesity, physical inactivity and modern lifestyle. Among them, obesity contributes in insulin resistance and abnormal glucose metabolism^1^. T2D can lead to many serious complications including cardiovascular, kidney and eye diseases and nerve damages as well. These diseases eventually may lead to premature death^2–4^. Moreover, treatment of T2D and its complications cost billion dollars per year for health care and also indirect costs caused by loss of productivity from disability and early mortality^5^. Accordingly, preventing or delaying the diabetes will improve the quality of individual life via reducing the diabetes-related complications. In recent years several various gene and protein biomarkers including retinol binding protein 4 (RBP4) have been introduced for prognosis of T2D^6^. RBP4 has been introduced as an independent predictive biomarker at early stages of insulin resistance^7^. The blood serum levels of RBP4 correlates significantly with other major factors related to insulin resistance such as triglyceride, cholesterol and blood pressure. Explaining the mechanism for the relation of elevated RBP4 with the risk of insulin resistance and T2D is complex due to the dark aspects of this process^8^. High fat storage in adipose tissues elevates the levels of RBP4 by which the expression of the insulin-stimulated glucose-transporter4 (GLUT4) in adipocytes is selectively reduced and consequently leads to decrease glucose uptake. In addition, increasing the level of RBP4 in blood serum might contribute to impair insulin-stimulated glucose uptake in muscle by decreasing enzyme PI-3 kinase activity. However, in liver, it can elevate hepatic glucose production by stimulating phosphoenol pyruvate carboxy kinase expression which is the characteristics of T2D^9^. An early study in 2014 has shown that RBP4 directly induces macrophages to secrete proinflammatory cytokines which results in greater inflammation in adipose tissue and insulin resistance^10^. Thus, these various effects of elevated level of RBP4 can lead to insulin resistance before any significant changes in diabetic markers in high risk population^11^. So, the elevation of RBP4 level can be a sign for catching the diabetes.

So far, various methods including ELISA (enzyme-linked immunosorbent assay) and conventional antibody-based assays have been presented for the quantitative measurement of human RBP4^12^. However, for improving specific detection and precise quantification of RBP4, aptamers were used. Aptamers are synthetic single-stranded oligonucleotides with high specificity and affinity to their targets that make them ideal candidates for molecular recognition systems^13^. Aptamers have been applied in biosensor design (aptasensor) due to their advantages over antibodies including: stability, availability, modifiability, flexibility and in some cases better specificity and affinity than similar antibodies^14^. RBP4 binding aptamer (RBA) is a 76-bp ssDNA sequence with very low Kd value (0.2 ± 0.03 µM)^15^. The specificity of this aptamer (RBA) has already been confirmed by using Vaspin, Nampt, adiponectin (ADPN), human serum albumin (HSA), human IgG and fibrinogen as negative controls^16^. RBA has also been applied in the enzyme-linked antibody-aptamer sandwich system with colorimetric reaction for more convenient and sensitive detection of RBP4 (78 ng/mL)^16^.

In the present study, an ultrasensitive aptasensor has been designed for the assessment of human RBP4 level in blood serum. In this system, RBP4 has been captured by immobilized RBA on plate and then detected by polyclonal luminol-antibody bearing intercross-linked gold nanoparticles (GNPs). GNPs were intercross-linked to each other via cysteine as a simple and low cost linker. Such big clusters increased the accessible surface for conjugating polyclonal antibodies and luminol molecules. Consequently, the efficient conjugation of polyclonal antibodies increased the probability of antibody-antigen interaction and on the other hand, the proficient conjugation of luminol molecules on GNP clusters could amplify the chemiluminescence signals. To our knowledge, there is no report applying RBA in chemiluminescent aptasensor. Application of chemiluminescence as a highly sensitive physical transducer together with the luminol-antibody bearing intercross-linked GNPs, will level up the aptasensor signal to an ultrasensitive scale.

## Results

### Characterization of GNPs

**Figure 1:**
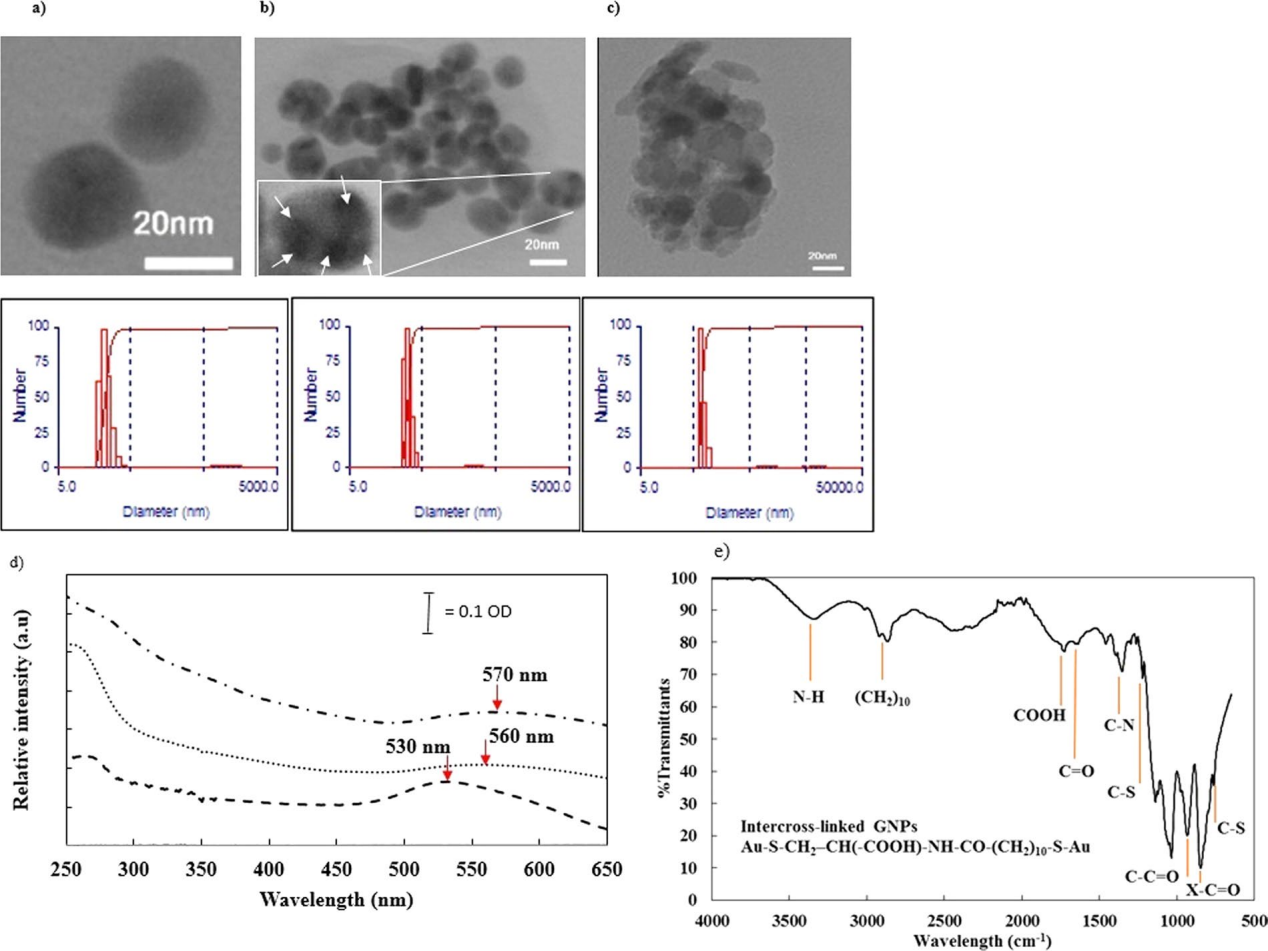
Transmission electron microscopy (TEM) images of three types of GNPs: (**a**) single GNPs and intercross-linked GNPs at (**b**) lower and (**c**) higher concentration of cysteine bearing GNPs. As shown in the lower part of images, the mean diameter obtained by DLS for (**a**, **b** and **c**) are 22.5, 31.7 and 67 nm, respectively. (**d**) UV-Visible spectroscopy of 20 nm-GNPs (Dashed line, Peak: 530 nm), cross-linked GNPs (Dotted line, Peak: 560 nm) and luminol-antibody bearing cross-linked GNPs (Dash-dotted line, Peak: 570 nm). (**e**) FT-IR spectrum of intercross-linked GNPs.

For single and intercross-linked GNPs characterization, the nanoparticle suspension was diluted in deionized water. The diameter of diluted single GNPs was measured by DLS to be in the range from 18 to 28 nm, while that for intercross-linked GNPs was in the range from 28 to 40 nm. These values were also confirmed by the images obtained by transmission electron microscopy (Fig. 1a–c).

The extinction coefficient (ε) for GNPs was calculated according to Eq. 1^17^:

1 $$\mathrm{Ln}\,{\rm{\varepsilon }}={\rm{k}}\,\mathrm{ln}\,{\rm{D}}+{\rm{a}}$$

where, D (nm) is the average diameters of the nanoparticles, k and a, were 3.32 and 10.8, respectively. Then, according to the OD_570_ calculated using the Eq. 2^17^, the single and intercross-linked GNPs concentrations were estimated to be 1.56 × 10^−6^ and 1.7 × 10^−9^ M, respectively:

2 $${\rm{O}}{\rm{D}}=\varepsilon {\rm{C}}$$

Intercross-linking of GNPs was also confirmed by using UV-Vis spectroscopy. In Fig. 1d, the absorption peak for GNPs (530 nm), intercross-linked GNPs (560 nm) and luminol-antibody bearing cross-linked GNPs (570 nm) have been compared. As seen after cross linking of GNPs the absorption peak was flattened and shifted to right owing to increase in particle size^18^. By immobilization of antibody and luminol on GNPs, the UV-Vis absorption peak was even shifted in some extend.

ATR-FTIR Spectroscopy was performed to characterize the intercross-linkage of GNPs. Since no characteristic absorption peak was observed for free -SH at 2559 cm^−1^, one may conclude that the free -SH groups in MUA and cysteine have already been bound onto GNPs surface. The characteristic peaks of –COOH vibrations at 1725 cm^−1^ and the saturated alkyl stretching vibration at 2867 and 2918 cm^−1^ were also indicated the presence of MUA onto GNPs surface. The peak at 1641 cm^−1^ (C=O stretching amides), the signal at 3400 cm^−1^ (–NH stretching amide) and the peak at 1350 cm^−1^ (C–N) prove the formation of (C–NH) amide bond via reaction between the primary amine groups of cysteine and the carboxylic groups of the MUA. The peaks at 760 and 1200 cm^−1^ correspond to the vibration of C–S bond which confirm the cysteine conjugation. These results indicate the formation of GNPs intercross-linked.

### Effect of pH and ionic strength on the sensor response

The ability of the aptasensor for RBP detection in a wide range of pH in PBS and carbonate buffers was investigated while the chemiluminescence signals were measured. The obtained data showed that the aptasensor reached to its maximum working sensitivity in 0.02 M PBS buffer at pH 8.0.

To optimize ionic strength, the sensor response was recorded in PBS containing different NaCl concentrations (50, 100,150, 200 mM). The maximum signal was obtained in 0.02 M PBS (pH 8) containing 100 mM NaCl.

### Signal amplification by intercross-linked GNPs

**Figure 2:**
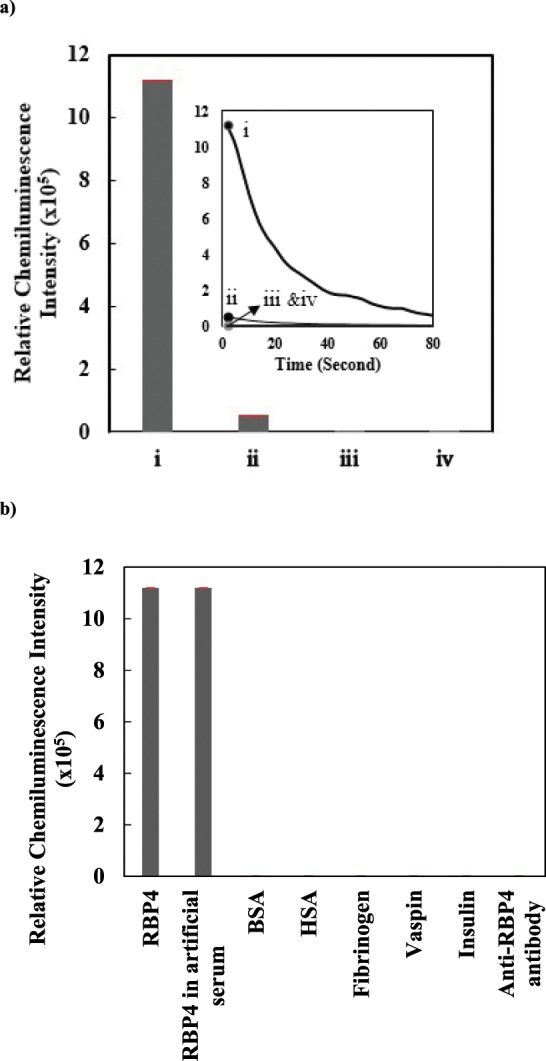
(**a**) Comparison between the chemiluminescence intensity obtained by aptamer/RBP4/luminol-antibody bearing intercross-linked gold nanoparticles (i) and aptamer/RBP4/luminol-antibody bearing gold nanoparticles (ii). In both tests the concentration of RBP4 was1 ng/mL. iii and iv are negative controls (Same as i and ii but, in the absence of RBP4) (**b**) Specificity of RBP4 biosensor towards different targets. Artificial serum was PBS containing 1 ng/mL of either main target (RBP4) or the other potentially interfering proteins: BSA, HSA, fibrinogen, insulin and anti-RBP4 antibody and vaspin.

In order to assess the ability of intercross-linked GNPs in signal amplification, the chemiluminescence intensity resulted from luminol-antibody bearing GNPs was compared with that obtained from luminol-antibody bearing intercross-linked GNPs. As shown in Fig. 2a, the intercross-linked GNPs exhibited more than 24 times chemiluminescence intensity relative to the GNPs.

**Figure 3:**
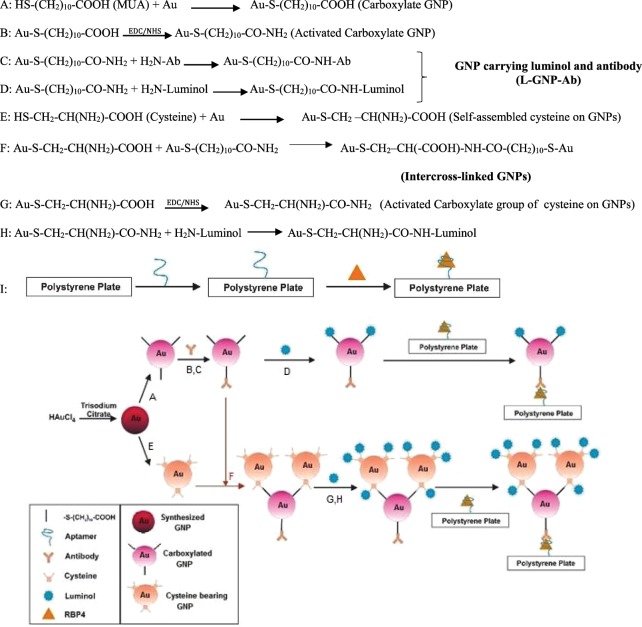
Steps for preparation of nano-aptasensor to monitor RBP4: (**A**) Carboxylation of GNPs via self-assembly of MUA on GNPs. (**B**) Activation of carboxylate functional group of GNPs, (**C**) Immobilization of antibody on GNPs (Ab-GNPs). (**D**) Immobilization of luminol on Ab-GNPs (L-GNP-Ab). (**E**) Self-assembly of cysteine on GNPs (Cys-GNPs). (**F**) Intercross-linking of Cys-GNPs and Ab-GNPs (GNP-Cys-GNP-Ab). (**G**) Activation of carboxylate functional group of cysteine on GNPs. (**H**) Immobilization of luminol on GNP-Cys-GNP-Ab. (**I**) Immobilization of biotinylated aptamer on streptavidin coated polystyrene micro well plate and then RBP4 protein binding to aptamer.

Application of luminol bearing nanoparticles for creating stable and strong signals is common in chemiluminesence biosensors. In the most of reports, antibodies are conjugated to luminol bearing GNPs covalently through the interactions between AuNPs and either mercapto or primary amine groups of biomolecules via Au-S and Au-N bonds. However, some research groups attempted to use the branched luminol bearing GNPs for increasing the sensitivity and enhancing the signal^19^. But in the present study, GNPs were intercross-linked to each other via Cys as a simple and low cost linker (Fig. 3F). As shown in Fig. 1c, at higher concentrations of cysteine, the GNPs intercrossed each other and formed the clusters with the diameters around 67 nm. Such big clusters increased the accessible surface for conjugating polyclonal antibodies and luminol molecules. Consequently, the efficient conjugation of polyclonal antibodies increased the probability of antibody-antigen interaction and on the other hand, the proficient conjugation of luminol molecules on the GNP clusters could amplify the chemiluminescence signals.

In 2005, Zhang *et al.* introduced a three steps mechanism for chemiluminescence signal production by luminol: oxidation of luminol to luminol radical and then oxidation of the luminol radical to a key intermediate followed by decomposition of hydroxyl hydroperoxide with the emission of chemiluminescence. Here we emphasize on the enhancement of chemiluminescence by GNPs in a luminol-H_2_O_2_ system. It is supposed that GNPs can facilitate the radical generation and then the electron-transfer process is taking place at the surface of GNPs^20,21^. Moreover, in our previous work^22^ it has been shown that gold even in ion form can act as an efficient catalyst for luminol oxidation and consequently increases the intensity of chemiluminescence signal. Entirely, all of these strategies resulted to creating extremely high chemiluminescence signals and increasing sensitivity to femto-gram level.

### Stability

The stability of luminol-antibody bearing intercross-linked GNPs was investigated by monitoring the chemiluminescence intensity of 10 μl of 0.5 nM GNPs in PBS (pH 8.0) solution during 21 days while the solution was kept at 4 °C in dark. Comparison of the chemiluminescence signals revealed that 95% of the initial intensity was retained after a 21-day storage period.

### Specificity

Figure 2b compares the specificity of aptasensor towards RBP4 and some abundant proteins in serum including BSA, HSA, fibrinogen, insulin and also anti-RBP4 antibody as an IgG and vaspin as a protein belonged to adipokines, as well. The chemiluminescent responses of aptasensor were recorded for a laboratory made serum containing 1 ng/mL of either the main target (RBP4) or other potentially interfering proteins (BSA, HSA, fibrinogen, insulin and anti-RBP4 antibody and vaspin). The results revealed that the proposed aptasensor responded more significantly to RBP4 than the other proteins. It seems that applying both aptamer and antibody improved the specific detection of RBP4. In addition, washing process in a sandwich-type procedure may have limited nonspecific binding, resulting in a low background signal. The specificity of RBA has also been previously confirmed by using Vaspin, Nampt. ADPN, HSA, human IgG and fibrinogen as negative controls^16^.

### Calibration curve

**Figure 4:**
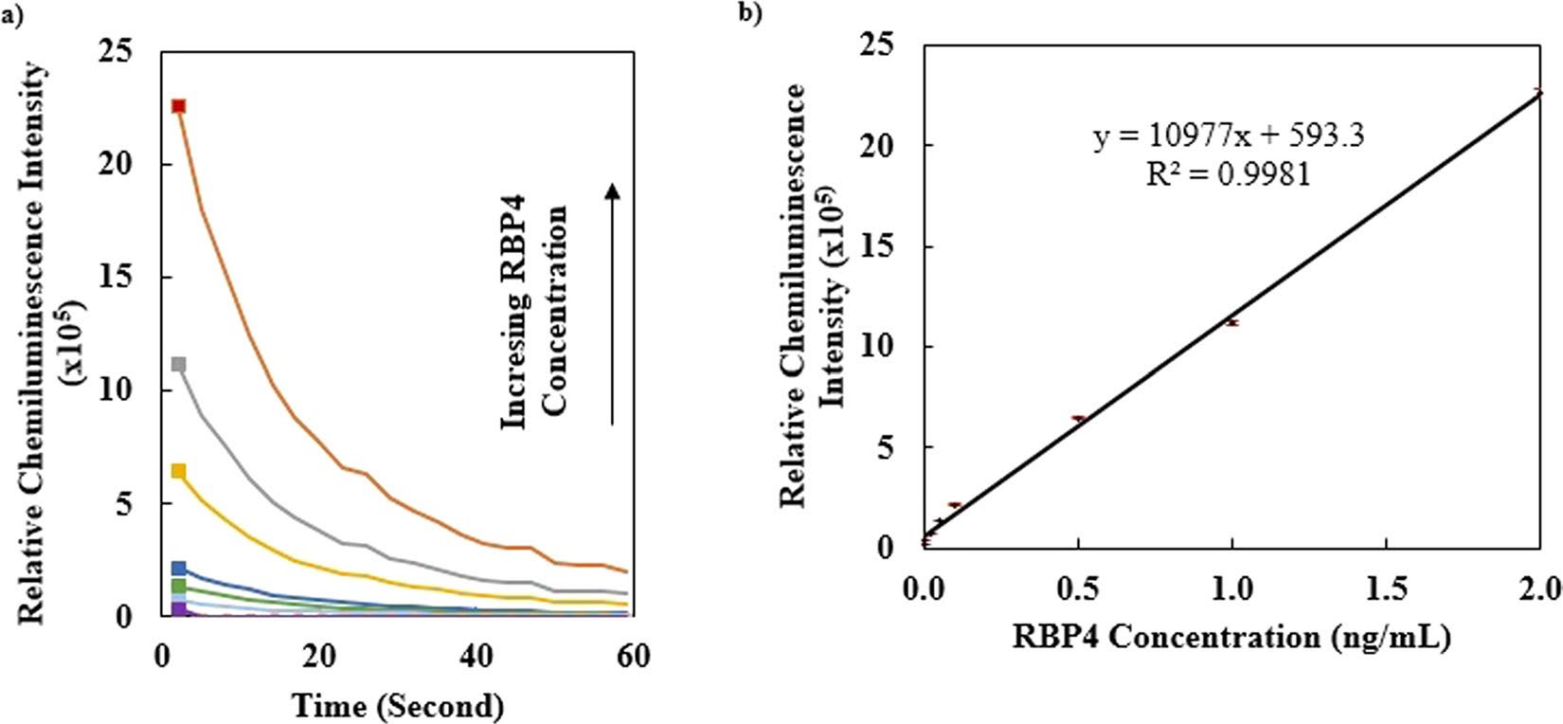
(**a**) Chemiluminescence intensity measurements by aptasensor in the presence of different concentrations of RBP4 (From down to up: 0, 0.001, 0.025, 0.05, 0.1, 0.5, 1 and 2 ng/mL). (**b**) Calibration curve for determination of RBP4. Each point stands for the mean value of three independent experiments.

Under the optimized conditions, the calibration curve for determination of RBP4 was plotted. As shown in Fig. 4, the chemiluminescence intensity increased linearly with increasing RBP4 concentration in the range from 0.001 to 2 ng/mL. According to Eq. 3, the detection limit was calculated to be less than 1 pg/mL (951 fg/mL), while the standard deviation at lowest concentration was 3.479. This value was lower than the detection limit for RBP4 obtained by other techniques (Table 1) except a recently reported biosensor which applying monoclonal antibody to detect RBP4 electrochemically^23^.

**Table 1:** Comparison between the parameters obtained by the present biosensor with those methods reported in the literature for determination of RBP4.

Detection Molecule	Detection method	Signal transduction	Linear range	Detection limit	Assay time	Reference
ssDNA Aptamer	Spectroscopy	Chemiluminescence	0.001–2 ng/mL	951 fg/mL	2 h	Present study
ssDNA Aptamer	Spectroscopy	Surface Plasmon Resonance (SPR)	0.2–0.5 μg/mL	1.58 μg/mL	2 h and 20 mim	^15^
ssDNA Aptamer	Spectroscopy	Enzyme-Linked Antibody-Aptamer Sandwich (ELAAS)	78 ng/mL to 5 µg/mL	75 ng/mL	2 h	^16^
Antibody	Spectroscopy	Enzyme-linked immunosorbent sandwich assay	1–3 ng/mL	>10 ng/mL	4 h and 20 min	^32^
Antibody	Electrochemical	Impedometric	0.01–1000 pg/mL	100 fg/mL	6 h	^23^
Antibody	Spectroscopy	ELISA	6.25–400 ng/mL	6 ng/mL	4 h	(abcam 108897)
Antibody	Mass sensitive	Matrix-Assisted Laser Desorption/Ionization Time-Of-Flight (MALDI-TOF MS)	7.81–500 μg/mL	3.36 μg/mL	46 min	^1^
Antibody	Mass sensitive	Quantitative Mass Spectrometry Immuno-affinity Assay (qMSIA)	7–500 μg/mL	—	46 min	^26^
Antibody	Spectroscopy	Chemiluminescence	1.33 ng/mL	0.62 ng/mL	1 h and 30 min	^33^
Antibody	Spectroscopy	Electro-chemiluminescence	78–5000 ng/mL	26 ng/mL	2 h	^34^

### Feasibility of aptasensor for real samples

The feasibility of present aptasensor for real samples was assessed by 20 serum samples from normal and diabetic individuals, obtained from Tehran University of Medical Sciences. At the same time RBP4 in real samples was determined by the aptasensor and Human ELISA Kit (ab108897) and the results were compared in Tables 2–3. The values of RBP4 concentrations in the samples were obtained by using the calibration curve (Fig. 4). By comparing the results of ELISA Kit with those obtained by aptasensor, a fine consistency was achieved. The intra- and inter-coefficient of variations (%CVs) were calculated to be less than 1%. This confirmed the reproducibility of aptasensor response toward RBP4.

**Table 2:** Determinations of RBP4 in real (normal and diabetic) female samples by the present aptasensor and ELISA kit.

Normal Female Samples	*Present Aptasensor	*ELISA µg/mL	Standard Deviation	%CV	Diabetic Female Samples	*Present Aptasensor	*ELISA µg/mL	Standard Deviation	%CV
1	21.738	21.733	0.004	0.018	1	34.893	34.888	0.004	0.011
2	23.73	23.76	0.021	0.088	2	37.001	37.002	0.001	0.002
3	29.9	29.913	0.009	0.03	3	31.353	31.348	0.004	0.012
4	27.1	27.101	0.001	0.003	4	35.46	35.463	0.002	0.005
Average	25.617	25.627	0.009	0.035	Average	34.677	34.675	0.002	0.005

The %CVs for intra- and inter-assay averages are 0.010 and 0.015, respectively.

*Mean of triplicate measurements.

**Table 3:** Determinations of RBP4 in real (normal and diabetic) male samples by the present aptasensor and ELISA kit. The %CVs for intra- and inter-assay averages are 0.010 and 0.015, respectively.

Normal Male Samples	*Present Aptasensor	*ELISA µg/mL	Standard Deviation	%CV	Diabetic Male Samples	*Present Aptasensor	*ELISA µg/mL	Standard Deviation	%CV
1	25.834	25.839	0.004	0.015	1	36.496	36.491	0.004	0.011
2	24.045	24.043	0.001	0.004	2	33.93	33.926	0.003	0.008
3	22.942	22.945	0.002	0.008	3	39.681	39.678	0.002	0.005
4	27.443	27.455	0.008	0.029	4	33.559	33.555	0.003	0.009
5	27.235	27.231	0.003	0.011	5	35.711	35.709	0.001	0.003
6	27.043	27.038	0.004	0.014	6	39.89	39.892	0.001	0.002
Average	25.693	25.759	0.004	0.015	Average	36.545	35.693	0.002	0.005

*Mean of triplicate measurements.

In addition, the experimental course for conventional ELISA takes about 3 hrs, while using the present biosensor this process would be don rapidly. Because in this approach the luminol-antibody bearing intercross-linked GNPs is prepared before starting the measurement. Moreover a simple and inexpensive laminator is used as detector of biosensor that record signals quickly. These features demonstrate the efficiency of biosensor for measuring RBP4 in real patient serums. Therefore, it seems that it has the potential to be applicable for commercial purposes.

## Discussion

Obesity and accumulative fat storage increase secretion of RBP4 from adipose tissues and plasma concentration. Elevating the level of RBP4 could be a sign for early detection of T2D^11^. In the present study, an ultrasensitive aptasensor for monitoring RBP4 at the detection limit of 951 fg/mL was designed and applied for predicting diabetes at early stages. Cysteine as an available, low-cost and easy to operate linker was used for intercross-linking of GNPs. By using the intercross-linked GNPs, it was possible to provide more accessible surface for immobilizing luminol and consequently amplifying the signal. Since the luminol-antibody bearing intercross-linked GNPs was prepared before starting the measurement, the aptasensor operation became relatively fast in comparison of the most previously reported detection systems. Detection of RBP4 was carried out by formation of an immune complex via a heterogeneous interaction between aptamer-RBP4-antibody which increases the specificity of sensor^24,25^. Moreover, a simple and inexpensive laminator was used as detector of biosensor that record signals quickly. As seen in Table 1, comparing to the biosensors reported in the literature, the analytical parameters such as sensitivity, specificity, detection limit, linear range and assay time were improved. Although, the assays based on mass spectrometry have been carried out in shorter time but this method is expensive and requires well-trained personnel which is not available in usual laboratories^1,26^. Therefore, the features demonstrated in Table 1 indicates the efficacy of aptasensor for RBP4 monitoring in real patient serums. As a result, it seems that the aptasensor has the potential to be applicable for commercial purposes, as well.

## Methods

### Materials and reagents

Luminol (5-amino 2,3-dihydro 1,4-phthalazinedione), H_2_O_2_ (30% solution) and three sodium citrate (Na_3_C_6_H_5_O_7_) were purchased from Merck (Germany). Gold (III) chloride hydrate (HAuCl_4_), bovine serum albumin (BSA), human serum albumin (HSA), Fibrinogen from human plasma, 1-ethyl-3-(3-dimethylaminopropyl) carbodiimidehydrochloride (EDC), N-hydroxysuccinimide (NHS), 11-mercapto undecanoic acid (MUA), 2-morpholinoethansulfonic acid monohydrate (MES), nonionic surfactant polyoxyethylene-20 sorbitanmonolaurate (Tween-20) and absolute ethanol were purchased from Sigma-Aldrich. RBA (76 mer ssDNA, 5ʹend) modified with biotin-T10 (10 times repeated thymidine bases) were provided by Metabion Inc., (Planegg/Steinkirchen, Germany). The sequence of RBA^15,16^ was: 5′-Biotin-TTTTTTTTTTATACCAGCTTATTCAATTACAGTAGTGAGGGGTCCGTCGTGGGGTAGTTGGGTC GTGGAGATAGTAAGTGCAATCT-3′. Human RBP4 protein (ab114060), anti-RBP4 antibody (ab114062) and RBP4 Human ELISA Kit (ab108897) were purchased from Abcam (Cambridge, USA) and the streptavidin coated polystyrene micro well were provided by Pishtaz Teb Zaman Co. (Tehran, Iran). All chemicals used without further purification. Also in all experiments the solutions were prepared using double distilled deionized water. All the instruments were set to zero with a blank solution to eliminate the interferer substances. A solution containing 150 mM NaCl, 25 mM Tris, 0.1% BSA and 0.05% Tween-20, pH 7.2, was prepared and used as washing solution. Another solution containing 100 mM NaCl, 20 mM Tris–HCl, 2 mM MgCl_2_, 5 mM KCl, 1 mM CaCl_2_ and 0.02% Tween-20, pH 7.6, was prepared and used as binding buffer.

### Apparatus

Dynamic light scattering (DLS, 90 plus Brookhaven Instruments Corporation, USA) was used for size determination. The single and intercross-linked GNPs images were obtained by PHILIPS CM30 Scanning Transmission Electron Microscope (Philips Electron Optics, Eindhoven, The Netherlands). For UV-Visible spectroscopy, the Cary 100 bio spectrophotometer (Varian, Australia) was used. Attenuated total reflectance-Fourier transform infrared spectroscopy (ATR- FTIR) was performed using Nicolet Nexus 470 FTIR (Nicolet, Madison, WI, USA) spectrometer. IrAnalyze software was also applied for IR spectrum interpretation (LabCognition, Ft. Myers, FL). TMicro centrifuge (Sigma, USA) was used to separate GNPs at different conditions. The samples were centrifuged at 14000 rpm for 30 min at 4 °C. The chemiluminescence emissions were recorded at 425 nm by fluorescence micro plate reader (H4, Bio Tech Co, USA).

### Synthesis of GNPs

GNPs were synthesized according to reference No.^27^. Based on the addition of different volume of 1% tri-sodium citrate to HAuCl_4_ solution, GNPs (Φ: 20–25 nm) were prepared. The resulting particle suspension stocked in a brown bottle at 4 °C.

### Preparation of intercross-linked GNPs

As shown in Fig. 3, two different types of modifications were carried out on GNPs: (i) process of A to C for preparation of single GNPs carrying luminol and antibody (L-GNP-Ab) and (ii) process of D to F for preparation of cross-linked GNPs carrying luminol and antibody (L-GNP-Cys-GNP-Ab).

During first process, carboxylated GNPs were prepared in such a way that 1 mL of GNPs was gently added to 2 mL of phosphate buffer solution (PBS, 10 mM, pH 8) containing MUA (3 mM) and Tween-20 (0.2 mg/mL) (Fig. 3A). The mixture was incubated at room temperature for 30 min. The thus prepared carboxylated GNPs were washed with buffer and centrifuged at 14000 rpm for 30 min. Following the careful removing of supernatant, containing excess MUA and Tween 20, the precipitate was re-suspended in buffer. For activation of the carboxylic acid functional groups assembled on GNPs, 1 mL of carboxylated GNPs was added to MES buffer solution (1 mM, pH 5.5) containing 50 μL of EDC (50 mM) and 50 μl of NHS (50 mM) and then, the mixture was incubated at room temperature for 20 min (Fig. 3B). Thereafter, the activated carboxylate GNPs were washed and centrifuged at 14000 rpm for 30 min three times. Finally, the precipitate was re-suspended in PBS. Then 25 μL of polyclonal antibody (20 mg/mL) was added to the activated carboxylate GNPs (Fig. 3C). For immobilizing luminol on antibody bearing GNPs, 200 μL of 10 mM luminol was added to this mixure and the mixture was incubated for 6 hrs at dark in cold room (at 4 °C) with gentle shaking. At the end, the mixture was washed and centrifuged at 14000 rpm for 30 min, three times (Fig. 3D). The prepared luminol-antibody bearing GNPs was diluted in PBS (0.01 M, pH 7.4) and stored at 4 °C.

During the second process, the intercross-linked L-GNP-Cys-GNP-Ab is prepared. For this purpose, according to reference No.^28^, cysteine was self-assembled on GNPs (Fig. 3E) and then, one mL of Cys-GNPs was added to two mL of Ab-GNPs. The mixture was incubated for 6 hours at dark in cold room (at 4 °C) with gentle shaking. At the end, the prepared intercross-linked GNPs (GNP-Cys-GNP-Ab) were washed and centrifuged at 7500 rpm for 30 min, three times (Fig. 3F). Finally, in order to conjugate the luminol molecules on intercross-linked GNPs, the free carboxylic acid groups either on cystein or carboxylated GNPs can be linked to amine groups of luminol molecules via EDC and NHS linkers (Fig. 3G). To conjugate the luminol molecules on intercross-linked GNPs, 200 μL of 10 mM luminol was added to 1.5 μL of intercross-linked GNPs and the mixture was incubated for 6 hours at dark in cold room (at 4 °C) with gentle shaking. At the end, the mixture was washed and centrifuged at 7500 rpm for 30 min for three times (Fig. 3H). The prepared L-GNP-Cys-GNP-Ab was diluted in PBS (0.01 M, pH 7.4) and stored at 4 °C.

### Immune complex formation and chemiluminescence measurements

As shown in Fig. 3I, streptavidin coated polystyrene micro well was used for biotinylated aptamer immobilization. Firstly, the wells were washed with 200 µL of washing solution and then, 100 µL of 0.4 µM biotinylated aptamer was added into each streptavidin coated polystyrene micro well and incubated for 2 h at room temperature while it was shaking mildly. To remove the non-immobilized reagents, the wells were washed three times. Then, a serial dilution of RBP4 protein in binding buffer was added to the wells^16^. After 1 h, the wells were washed and aptamer-target complexes were incubated with 100 μL of either L-GNP-Ab or L-GNP-Cys-GNP-Ab, while shaking at 25 °C in dark for 1 h. Finally, the wells were washed with 200 μL of washing solution in dark.

The response of aptasensor in PBS containing different concentrations of NaCl were investigated. For chemiluminescence measurements, optimum PBS buffer (pH 8, 0.02 M containing 100 mM NaCl) was added to the immune complex and then HAuCl_4_ (10 μl, 0.1% w/v) as catalyst and H_2_O_2_ (10 μL, 10^−2^ M) as initiator were injected to the solution and the chemiluminescence intensity was recorded. Then calibration curve was plotted based on chemiluminescence intensities against the serial dilution of RBP4 protein. Then detection limit (DL) was calculated according to Eq. 3^29^:

3 $${\rm{DL}}=3\,{{\rm{S}}}_{{\rm{a}}}/{\rm{b}}$$

where, S_a_ is the standard deviation of the response and b is the slope of the calibration curve.

### Feasibility of aptasensor for real samples

The feasibility of present aptasensor for real samples was assessed by 20 serum samples from normal and diabetic individuals, obtained from Tehran University of Medical Sciences. At the same time RBP4 in real samples was determined by the aptasensor and Human ELISA Kit (ab108897) and the results were compared. To verify the reproducibility of responses, the coefficient of variation (%CV) was calculated. First, the standard deviation (σ) was calculated according to Eq. 4^30^:

4 $$\sigma =\sqrt{\frac{\sum (Result-Mean{)}^{2}}{N-1}}$$

To analyze intra-assay CV, three replicates of three different samples were run in one assay. To analyze inter-assay CV, three different samples were run in three independent assays. The inter or intra-assay variation (%CVs) was calculated using Eq. 5^31^:

5 $$ \% {\rm{CVs}}=\sigma /({\rm{mean}}\,{\rm{of}}\,{\rm{assay}}\,{\rm{results}})\times 100$$

The values of %CVs less than 10 and 15, respectively for intra-assay and inter-assay, are generally acceptable.

### Ethical approval and informed consent

This study complied with the Declaration of Helsinki (1989 revision) and was approved by the Ethics Committee of the University of Tehran. All of the participants signed an informed consent form before enrollment in the study. All methods and experiments were performed in accordance with the standard ethical guidelines and approved by Ethics Committee of the University of Tehran.
